# The LKB1 Tumor Suppressor as a Biomarker in Mouse and Human Tissues

**DOI:** 10.1371/journal.pone.0073449

**Published:** 2013-09-25

**Authors:** Yuji Nakada, Thomas G. Stewart, Christopher G. Peña, Song Zhang, Ni Zhao, Nabeel Bardeesy, Norman E. Sharpless, Kwok-Kin Wong, D. Neil Hayes, Diego H. Castrillon

**Affiliations:** 1 Department of Pathology and Simmons Cancer Center, UT Southwestern Medical Center, Dallas, Texas, United States of America; 2 Departments of Medicine and Genetics, The Lineberger Comprehensive Cancer Center and University of North Carolina, Chapel Hill, North Carolina, United States of America; 3 Department of Clinical Sciences, UT Southwestern Medical Center, Dallas, Texas, United States of America; 4 Massachusetts General Hospital Cancer Center and Harvard Medical School, Cambridge, Massachusetts, United States of America; 5 Department of Medicine, Harvard of Medical School and Dana Farber Cancer Institute, Boston, Massachusetts, United States of America; Centro Nacional de Investigaciones Oncológicas (CNIO), Spain

## Abstract

Germline mutations in the *LKB1* gene (also known as *STK11*) cause the Peutz-Jeghers Syndrome, and somatic loss of LKB1 has emerged as causal event in a wide range of human malignancies, including melanoma, lung cancer, and cervical cancer. The LKB1 protein is a serine-threonine kinase that phosphorylates AMP-activated protein kinase (AMPK) and other downstream targets. Conditional knockout studies in mouse models have consistently shown that LKB1 loss promotes a highly-metastatic phenotype in diverse tissues, and human studies have demonstrated a strong association between LKB1 inactivation and tumor recurrence. Furthermore, LKB1 deficiency confers sensitivity to distinct classes of anticancer drugs. The ability to reliably identify LKB1-deficient tumors is thus likely to have important prognostic and predictive implications. Previous research studies have employed polyclonal antibodies with limited success, and there is no widely-employed immunohistochemical assay for LKB1. Here we report an assay based on a rabbit monoclonal antibody that can reliably detect endogenous LKB1 protein (and its absence) in mouse and human formalin-fixed, paraffin-embedded tissues. LKB1 protein levels determined through this assay correlated strongly with AMPK phosphorylation both in mouse and human tumors, and with mRNA levels in human tumors. Our studies fully validate this immunohistochemical assay for LKB1 in paraffin-embedded formalin tissue sections. This assay should be broadly useful for research studies employing mouse models and also for the development of human tissue-based assays for LKB1 in diverse clinical settings.

## Introduction

*LKB1* (also known as *STK11*) has emerged as a major tumor suppressor in diverse malignancies, particularly melanoma, cervical cancer, and lung cancer [1]–[5]. The *LKB1* gene encodes a serine/threonine kinase that acts through a multitude of targets to control diverse aspects of cell polarity, metabolism, and cell growth [6], [7]. Among these diverse substrates, the α catalytic subunit of AMP-activated protein kinase (AMPK) is the best established both in normal physiologic states and cancer. The LKB1 protein, in association with the accessory proteins STRAD and MO25, phosphorylates AMPKα at Thr172 in its activation loop, leading to AMPK activation when AMPK is in the AMP-bound state. AMPK directly phosphorylates TSC2 and raptor to suppress signaling through mTOR pathway, and mTOR pathway hyperactivity in the LKB1-deficient state is believed to account for some, but not all of LKB1 tumor suppressor functions [8], [9].

Considerable preclinical evidence exists that LKB1 deficiency confers an unusually poor clinical outcome, sensitivity to distinct classes of anticancer drugs, such as mTOR and SRC inhibitors, and resistance to other drug classes, such as MEK inhibitors [10]–[14]. It appears very likely that the ability to reliably identify LKB1-deficient tumors would have ‘theranostic’ implications, and thus be of clinical utility. However, no LKB1-based clinical assay has been developed since the identification of LKB1 as a tumor suppressor in 1998 [15]. In general, it has proven much more difficult to develop useful clinical assays based on tumor suppressor inactivation (e.g. deletion of *RB or PTEN*) than oncogene activation (e.g. *ERBB2* amplification, *RAS*/*RAF* activation). In large part this relates to the much more expanded set of molecular alterations that can inactivate a tumor suppressor (point mutation or intragenic deletions throughout coding and *cis*-regulatory regions, promoter hypermethylation, or diverse post-translational modifications leading to protein instability) vs. the more limited set of molecular alterations that define oncogene activation. This has made it difficult to develop clinical assays with sufficient specificity to reliably define states of tumor suppressor loss. For example, even whole-gene sequencing has high false negative rates as it misses several mechanisms that frequently inactivate tumor suppressor proteins (e.g. degradation, promoter hypermethylation, etc.).

Another practical consideration that further complicates such efforts–particularly DNA-based assays– is that most tumors contain abundant “contaminating” cells (e.g. lymphocytes, tumor-associated stroma), which, in many cases, outnumber the malignant cells harboring the actionable molecular alterations. Specific gain-of-function/oncogenic mutations or gene amplification events are readily detectable even in tumors with an abundant background of such contaminating cells, whereas loss-of-function mutations and deletions are more easily obscured.

In principle, the ability to reliably detect a tumor suppressor protein *in situ* presents an attractive alternative, particularly as it has the potential to capture most of the above mechanisms, including post-translational mechanisms conferring protein instability. Several factors make LKB1 a particularly appealing candidate for such efforts. First, intragenic deletions, insertions, and splicing mutations (leading to frameshifts with alteration of epitopes or complete absence of protein) are very common in *LKB1*, as are larger intragenic deletions ranging from a single exon up to 100 kb of genomic DNA [1]. Taken together, these types of mutations occur in at least 50% of cases harboring *LKB1* mutations [16]. Furthermore, some *LKB1* point mutations result in decreased protein stability [17]. However, identification of an antibody with sufficient sensitivity and specificity suitable for *in situ* detection is often difficult, and validation itself can be challenging particularly if the tumor suppressor is ubiquitously expressed, as is usually the case.

Although there are reports of LKB1 immunodetection in specific research settings, no assay has been extensively validated, proven robust, or widely adopted; e.g. across multiple cancer types [11], [18]–[22]. Here we describe a rabbit LKB1 monoclonal antibody capable of detecting the endogenous protein in clinical material (i.e. paraffin-embedded, formalin-fixed human tissue) with excellent performance characteristics. Furthermore, this method is also applicable to studies of LKB1 loss in murine preclinical model systems. Additional studies demonstrated that assays based on this approach can serve as the basis of clinical tests to identify tumors characterized by LKB1 loss.

## Materials and Methods

### Ethics Statements

Mouse experiments were conducted with the approval of the UT Southwestern Institutional Animal Care and Use Committee and the Dana Farber Institutional Animal Care and Use Committee. For the human biomarker studies, study subjects were accrued as part of a research protocol (requiring written informed consent) approved by the University of North Carolina Office of Human Research Ethics (see also below).

### Mouse colonies, alleles, and Adeno-Cre virus instillation

Mice were housed in a pathogen-free animal facility in microisolator cages and fed *ad libitum* on standard chow under standard lighting conditions; experiments were conducted with the approval of Institutional Animal Care and Use Committees. *Sprr2f-Cre*; *Lkb1^L/L^* endometrial Lkb1-knockout mice were bred and generated as previously described [10]. Breeding of *LSL-Kras^G12V^*; *Lkb1^L/L^* mice and Adeno-Cre nasal instillation to effect bronchial Cre-mediated recombination was conducted as previously described [2]. Mice were treated with Adeno-Cre at 8 weeks of age and euthanized 10 weeks later.

### Cell lines and preparation of cell blocks for immunohistochemistry

Human cervical carcinoma cell lines HeLa [cat# CCL-2] and CaSki [cat# CRL-1550] were purchased from the ATCC and grown on plastic tissue culture plates in low glucose DMEM (Gibco) +10% fetal bovine serum. Tet-On-LKB1-Hela cells (see below) were grown in low glucose DMEM (Gibco) +10% tetracycline-free fetal bovine serum (Clontech) + puromycin (1 µg/ml) (Clontech) and G418 (400 µg/ml) (Gibco) media. To overexpress human LKB1 in these cells a Lentivirus-Tet-On-LKB1 plasmid was generated by cloning an *LKB1* cDNA into the BamHI-XbaI sites of the pLVX-Tight-Puro vector (Clontech). The pLVX-Tight-Puro-LKB1 and the pLVX-Tet-On Advanced vectors (Clontech) were co-transfected (1∶1 ratio) with the Lenti-X HT Packaging Mix kit (Clontech) into HEC293T cells. 24 hours later, the transfection medium was replaced with fresh medium and incubated at 37°C for 48 hours to produce lentivirus particles. HeLa cells were infected with Lentivirus-Tet-On-LKB1 particles for 24 hours with polybrene (4ug/ml) (Sigma-Aldrich H9268) followed by replacement of the culture medium with fresh complete culture medium. To induce LKB1 expression, doxycycline (500 ng/ml) (Sigma-Aldrich) was added to the culture medium and incubated at 37°C for 48 hours.

Confluent cells were harvested with a cell scraper without trypsin/EDTA treatment and briefly spun down in a 15 ml conical tube. The cell pellet was resuspended in 10% buffered formalin and incubated at RT for 1 hour. The cells were washed twice in PBS, resuspended in an equal volume of 2% low melting temperature agarose (Cambrex), and cast in the wells of 96-well plates. To simulate routine clinical pathology laboratory processing of diagnostic tissue samples, the solidified plugs were subjected to overnight fixation and paraffin-embedding, and cut into 5 µ sections.

### Tissue processing, LKB1/pAMPKα (Thr172) immunohistochemistry, and scoring of protein levels

Tissues were fixed in 10% formalin for 24 hours at 4^o^, washed twice in PBS, then processed and embedded in paraffin. 5 µ sections were cut onto SuperFrost Plus slides (Fisher Scientific), deparaffinized in xylene, and hydrated in a graded ethanol series. Antigen retrieval was performed by gentle boiling in 10 mM sodium citrate pH 6.0 followed by cooling at room temperature for 20 minutes. Endogenous peroxidase activity was quenched with 3% hydrogen peroxide in ddH_2_0 for 30 minutes, followed by blocking in 1% BSA (in PBS) for 15 minutes. The primary antibodies used for immunohistochemistry were α-phospho-AMPKα (Thr172) (1∶50 dilution) (rabbit monoclonal 40H9, Cell Signaling Technologies catalog #2535) and a concentrated preparation of α-LKB1 rabbit monoclonal D60C5 (Cell Signaling Technologies #3047BF [2.2 mg/ml in PBS]), used at dilutions of 1∶10000 (for mouse tissues) and 1∶500 (for human tissues) of ). Note: this is available as a custom reagent from the manufacturer. The “off-the-shelf” manufacturer's preparation (catalog #3047) is provided at a concentration optimized for Western blotting that is too low for immunohistochemistry of human tissue sections; i.e. this reagent (24 µg/ml, Cell Signaling Technologies #3047) would require 1∶6 dilution. We recommend that each laboratory optimize antibody dilutions for every tissue. The other commercial antibodies tested on tissue sections of our paraffin-embedded, formalin fixed human cell line panel and *Sprr2f-Cre*; *Lkb1^L/L^* mosaic uterus were: Proteintech rabbit polyclonal (catalog #10746); Cell Signaling rabbit monoclonal 27D10 (cat#3050s); EMD Millipore rabbit polyclonal (catalog# ST1092); EMD Millipore mouse monoclonal 5C10 (catalog #05-832); Abcam mouse monoclonal Ley37D/G6 (catalog #ab15095). These antibodies were tested using the above immunodetection protocol at titers ranging from 1∶100 to 1∶10000 with no evidence of specific staining at any titer (see results for representative example-Ley37D/G6).

ImmPRESS (Vector laboratories) was employed as a secondary detection system, and applied for 30 minutes. For detection, DAB (3,3′-diaminobenzidine) (Dakocytomation) was used as a substrate-chromogen. The processed slides were counterstained with hematoxylin, air-dried, and mounted in Permount (Fisher Scientific).

Lung cancer tissue cores in a triplicate set of tissue-microarrays (TMAs) were used to collect immunohistochemistry data. Based on the range of staining intensities observed on the TMAs, investigators scored LKB1 protein expression on each slide with a four category scale. The total signal within the cell was scored with no distinction between subcellular localization patterns, since no obvious variation in subcellular expression patterns (e.g. in nuclear vs. cytoplasmic localization) were noted. Only cancer cells were scored; tumor stroma was not evaluated. Stromal staining was evident in most cores, serving as a useful positive control and reference point for staining intensity. The maximum score of the triplicate set was selected as a single summary value for the entire set. Patients were included in each analysis for which complete clinical, genetic, and immunochemistry data was available.

### Patient Clinical and Genomics Data

Study subjects were accrued as part of a tissue banking protocol under Institutional Review Board approved protocols 90-0573 and 07-0120 at UNC. All patients were treated for non-small cell lung cancer at UNC hospitals and clinical data was obtained by retrospective chart review. All genomic data (gene expression arrays, copy number array, and sequencing) from the current study has been previously reported and are publically available and are described in detail elsewhere [23]–[25]. Gene expression data is from the Agilent 44 K platform to measure gene expression, copy number data was obtained using the Affymetrix Mapping 250 K Sty2 SNP Array and the Affymetrix Genome-Wide Human SNP 6.0 Array. Sequencing of *LKB1* has been described in the prior report [24].

### Statistical methods

Ordered logistic regression was employed for analysis in cases of ordered categorical outcomes. For group comparisons the Wilcoxon rank sum statistic was used. For differences in categorical outcomes Fisher's exact test was used. All analyses and figures were performed using the R 2.15.2 software environment (21).

## Results and Discussion

**Figure 1:**
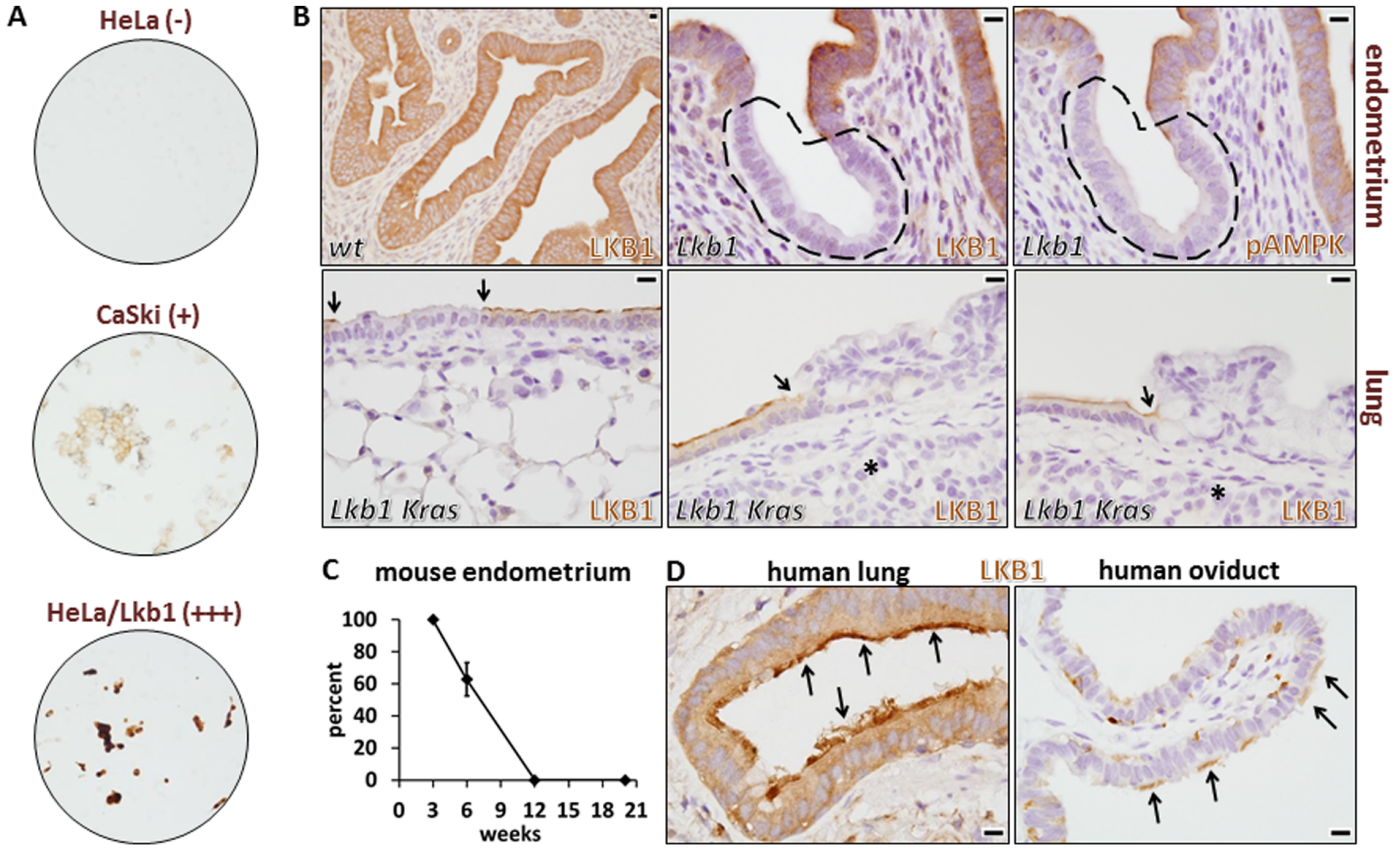
Validation of LKB1 rabbit monoclonal antibody D60C5 for immunohistochemistry in human and mouse paraffin-embedded, formalin-fixed samples. A, Human cervical cancer cell lines. HeLa/Lkb1  =  HeLa cells following transduction of lentivirus harboring a human *LKB1* cDNA inducible expression construct. Relative expression levels of Lkb1 protein are indicated in parentheses. B, Mouse tissues (endometrium and lung) from animals harboring floxed alleles of *Lkb1* following Cre-mediated recombination with *Sprr2f-Cre* (endometrium) or nasal-instillation of Adeno-Cre virus (lung). Distinct Lkb1-null clones are indicated by dashed lines (endometrium) or arrows (lung). Bars = 10 µm for each panel. Asterisks in the lung panels show invasive cancer cells subjacent to the dysplastic epithelium; these invasive cancer cells are also clearly Lkb1-null. C, Percent of Lkb1-null cells in *Sprr2f-Cre*; *Lkb1^L^* ^/*L*^ female mice by immunohistochemistry at 3, 6, 12, and 20 weeks of age. Error bar  =  S.E.M. D, Normal patterns of LKB1 protein in human lung and oviduct highlighting localization to the apical surface of ciliated cells (arrows). Note: in the oviduct, ciliated epithelial cells (arrows) are interspersed among nonciliated cells. Bars = 10 µm in both panels.

To screen for a suitable antibody, we analyzed a panel of three human cancer cell lines: HeLa (harbors biallelic *LKB1* deletions, expresses no protein), CaSki (*LKB1* wild-type, expresses normal levels of protein), and HeLa transduced with an *LKB1* cDNA (expresses abnormally high levels of protein). The cells were grown under routine culture conditions, and subjected to formalin-fixation and paraffin-embedding to simulate clinical conditions. Five commercial (see methods) and several non-commercial α-LKB1 antibodies were comprehensively tested, including two rabbit monoclonals and two mouse monoclonals. Results for only one antibody (rabbit monoclonal clone D60C5) were consistent with faithful binding to and detection of the LKB1 protein by standard indirect immunohistochemistry (Fig. 1A). These analyses are detailed below.

As previously described, by 12 weeks of age, *Lkb1* loss drives endometrial cancer in mice, while combined *Lkb1* loss/*Kras* activation drives the formation of lung cancer. The endometrial-specific *Sprr2f-Cre* driver results in Cre-mediated recombination in 50–60% of endometrial epithelial cells by 6 weeks of age, providing an ideal mosaic system for *in vivo* biomarker validation [10]. In wild-type murine endometria, all epithelial cells stained strongly and uniformly for Lkb1 (Fig. 1B). Stromal cells also expressed the protein, albeit at lower levels. In sharp contrast, however, *Sprr2f-Cre*; *Lkb1^L/L^* mice at 6 weeks of age exhibited a strikingly mosaic pattern of Lkb1 expression, consistent with clonal Cre-driven loss of *Lkb1* and specific Lkb1 immunodetection. Of note, under optimized conditions, background staining in the Lkb1-null epithelial clones was minimal. Concordantly, pAMPKα (Thr172) immunohistochemistry of serial sections provided compelling evidence that 1) the pAMPKα (Thr172) antibody was also reliable for *in situ* analyses and 2) AMPKα is indeed an Lkb1 target hypophosphorylated *in vivo* following Lkb1 loss (Fig. 1B). At 12 and 20 weeks, 100% of *Sprr2f-Cre*; *Lkb1^L^* ^/*L*^ endometrial epithelial cells were Lkb1-null, consistent with a selective growth advantage of the Lkb1-null cells (Fig. 1C).

**Figure 2:**
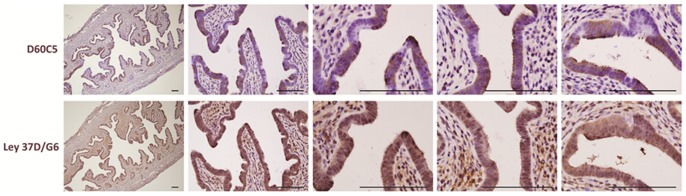
Testing of another α-LKB1 monoclonal antibody (Ley 37D/G6). Tissue sections are from the uterus of a 6-week old *Sprr2f-Cre*; *Lkb1^L^* ^/*L*^ female mouse. Rabbit monoclonal D60C5 readily distinguishes LKB1 positive from negative cells as shown previously. In contrast, the mouse monoclonal antibody Ley 37D/G6 shows a homogeneous pattern throughout the endometrial epithelium (serial step section) and fails to distinguish between LKB1 positive and negative cells. Size bars = 100 µ for each panel.

Similar results were obtained in an *Lkb1*/*Kras* adeno-Cre nasal instillation model of lung carcinogenesis [2]. Whereas control lungs showed uniform Lkb1 expression, mice treated with adeno-Cre showed mosaic patterns of Lkb1 expression in the bronchial epithelium that is subject to adenovirus infection. Furthermore, while some Lkb1-deficient clones appeared morphologically normal, most showed clear evidence of dysplasia and hyperplasia (leftmost vs. two right lung panels, Fig. 1B), consistent with a causal association between Lkb1 loss and dysplasia/tumor progression. Invasive tumor cells in this model were always Lkb1-negative (asterisks, Fig. 1B). Interestingly, in ciliated cells (lung bronchial epithelium, oviductal epithelium), the LKB1 protein was prominently expressed in the apical surface (i.e. in the cilia), consistent with LKB1's known roles in the establishment and maintenance of epithelial polarity [26]. This asymmetric staining also serves as a convenient positive control whereby these readily-available normal human tissues can be used to confirm that immunostaining was performed correctly (Fig. 1D). We conclude that this LKB1 antibody reliably detects the endogenous protein in diverse human and mouse tissues, making it ideal for biomarker studies employing both human cancer specimens (see below) and mouse cancer models. For comparative purposes, a representative monoclonal antibody (Ley37D/G6) for which we could not demonstrate specific immunostaining in either the human cell line panel or in Lkb1-mosaic mouse tissues is shown (Fig. 2). We note that our inability to demonstrate specific immunostaining under our experimental conditions does not invalidate the potential utility of an antibody for specific experimental conditions, tissues, etc., that we did not test.

**Figure 3:**
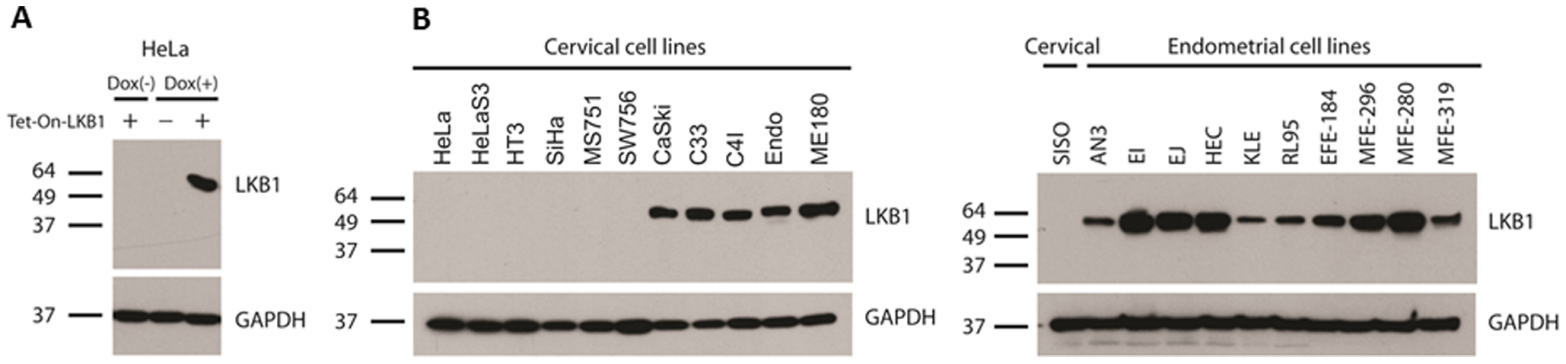
Validation of rabbit monoclonal antibody D60C5 by Western blotting. Positions of molecular weight standards (kilodaltons) are shown to the left of each blot. A, HeLa cells harboring Tet-On construct inducible with doxycycline. B, Comprehensive uterine cancer cell line panel (endometrial and cervical). Note: C4I harbors biallelic mutations of LKB1: a chromosomal deletion plus a point mutation that does not affect protein levels [1]. CaSki, C33, and ME180 do not harbor LKB1 mutations [1]. Endo was derived from normal endocervical epithelium immortalized with HPV E6/E7 [27].

Western blotting was performed to further validate and test the specificity of this antibody. In lysates obtained from unmodified HeLa cells or HeLa cells harboring a Tet-on-LKB1 construct prior to induction, no protein species were detected. Following induction with doxycycline, however, a specific band corresponding to LKB1 was detected (Fig. 3A). To further test the performance of the antibody, it was also tested against lysates derived from a comprehensive panel of uterine cell lines, both endometrial and cervical (n = 22). No LKB1 protein was detected in most cervical cancer cell lines (6/11), consistent with prior reports that a high percentage of cervical cancers are characterized by biallelic *LKB1* inactivation. However, LKB1 was expressed in some cervical cancer cell lines, as previously reported [1]. LKB1 protein was expressed in all endometrial cancer cell lines (n = 10), although there was considerable variation in its expression levels (Fig. 3B). Note that all of these images were minimally cropped and no other species were detected. These results demonstrate that the antibody is essentially monospecific with respect to LKB1 when tested against a diverse panel of human cell lines by Western blotting. We conclude that this antibody displays exceptional specificity for LKB1.

**Figure 4:**
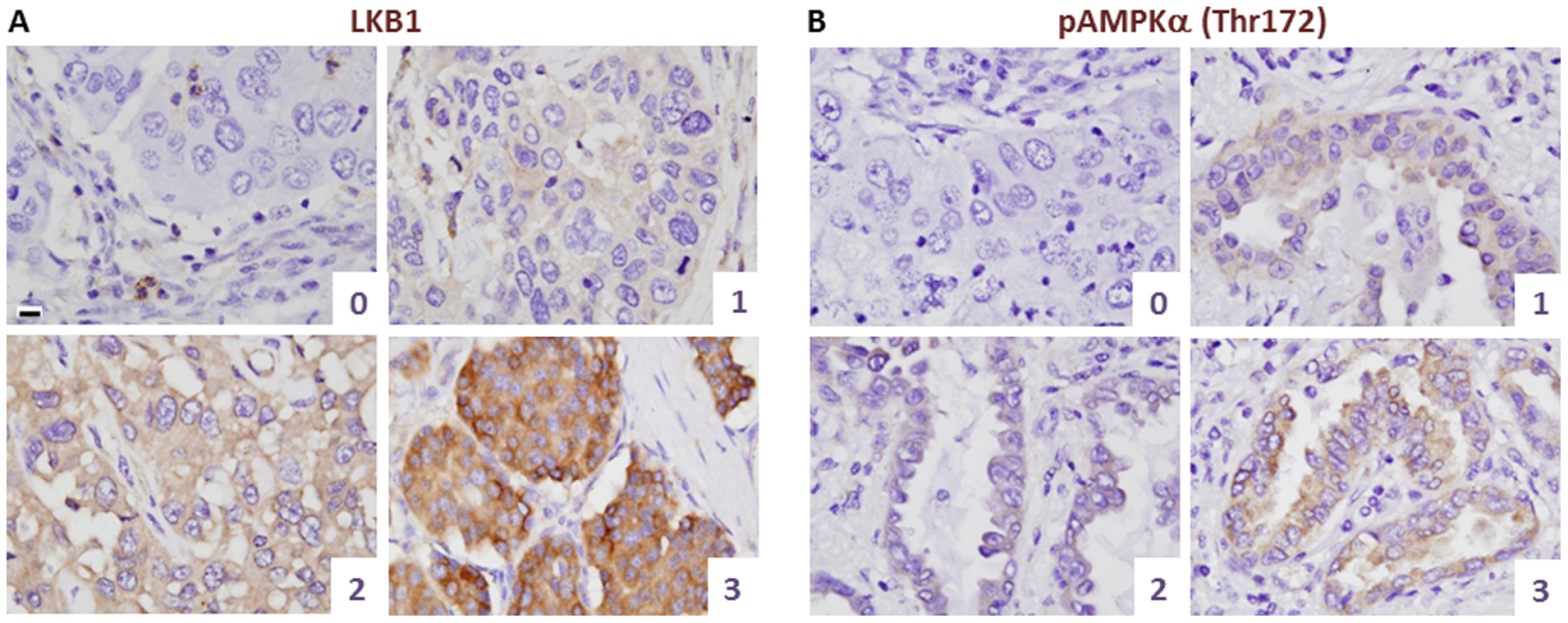
Scoring schema for LKB1 and pAMPKα (Thr172) expression in human lung cancer specimens. Tissues were paraffin-embedded and fixed in formalin. Only staining in the malignant epithelial cells was scored. A, LKB1 immunohistochemistry and representative cases illustrating histologic scores. B, pAMPKα (Thr172) immunohistochemistry and representative cases illustrating histologic scores. The dynamic range was somewhat lower for pAMPKα (Thr172) vs. LKB1 but a wide range of staining intensities was also observed. Bar = 10 µm in all panels; all panels are at same magnification.

To test the utility of the antibody in clinical lung cancer specimens, immunohistochemistry was performed on a triplicate set of tissue-microarrays (TMAs) containing lung cancer tissue cores. Patient demographics were representative of a typical clinical population in the USA (Table 1). Interestingly, a very broad range of LKB1 staining intensities were observed, showing that LKB1 levels are highly variable in lung cancers, and hence that LKB1 could serve as a discriminating biomarker. Based on the range of staining intensities observed, a scoring scale (0–3) was devised, where 0 = no appreciable staining; 1 = very low staining but above background; 2 = strong staining; and 3 = very strong staining. Each member of the triplicate set was scored, and the maximum score was selected as a single summary value for the entire set. In general, staining intensities in each tissue core were uniform across the tumor cells (Fig. 4A). To further validate LKB1 as a potential biomarker, TMA slides were also stained for pAMPKα (Thr172) and a similar scoring scheme was devised (Fig. 4B). A total of 123 cases were scored for both markers (Table 1).

**Table 1:** Summary of patient data.

Variable	Percent	Count
Sex		
Female	49%	60
Male	51%	63
Race		
Asian	2%	2
Black	20%	24
White	79%	97
Ethnicity		
Hispanic	1%	1
Not Hispanic	99%	122
Smoking Status		
Current Smoker	44%	54
Former Smoker	48%	59
Never/Light Smoker	8%	10
Histology		
Adenocarcinoma	56%	69
Squamous	36%	44
Large Cell	6%	7
Other	2%	3
Median Age at Diagnosis	65.8 years	

**Figure 5:**
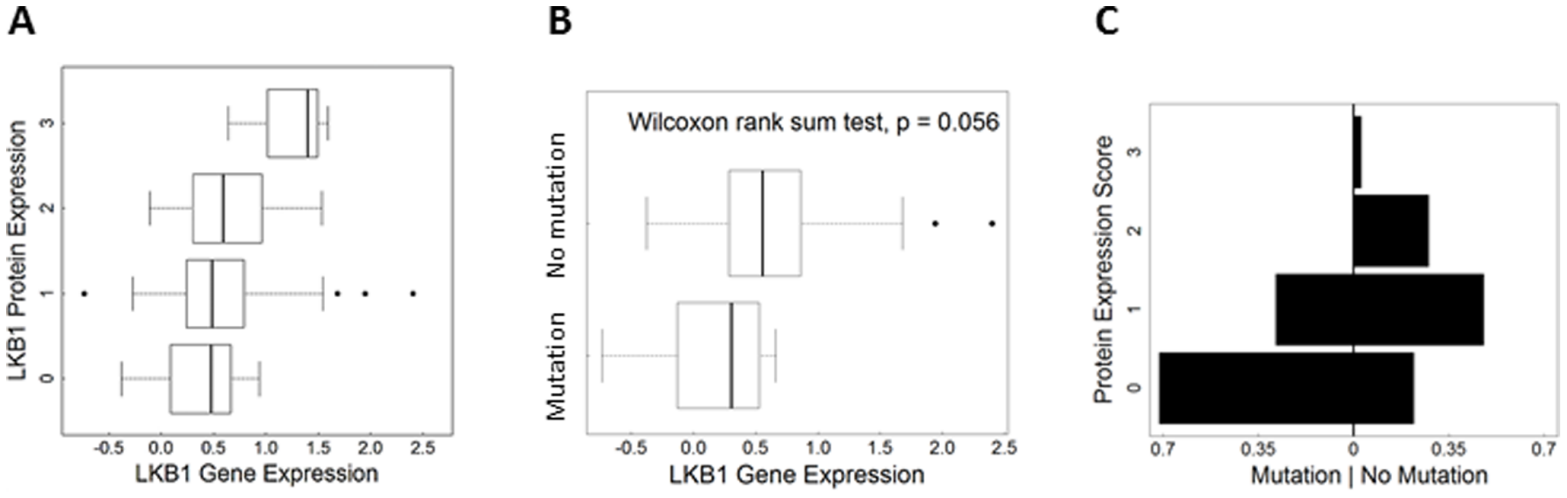
Validation of LKB1 antibody *in situ* assay in human lung tumor specimens. A, LKB1 protein expression vs. gene expression scores. Researchers used the Agilent 44(p<0.01). B, Box plots showing comparison of gene expression scores in cases with confirmed *LKB1* loss-of-function mutations vs. cases with no mutations. C, Protein expression by mutation status providing visual comparison of cases with mutation vs. no mutation. The x-axis shows the percentage of cases per LKB1 score (i.e. each side adds up to 1). The unsymmetrical shape indicates differences between the groups.

**Figure 6:**
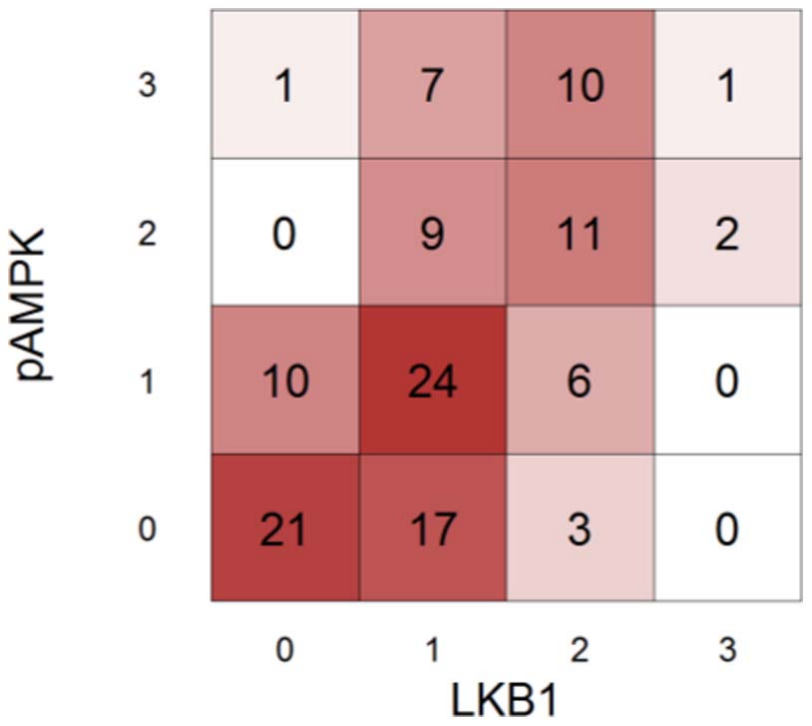
Correlation between LKB1 and pAMPKα (Thr172) scores. Heat map shows associations between LKB1 and pAMPKα (Thr172) protein expression scores. Kendall's tau provides a summary of the correlation (τ_κ_ = 0.49, p<0.001).

We then assessed relationships between LKB1 protein expression in this TMA of human lung cancers and other genomic measures performed on RNA/DNA prepared from fresh samples of the same tumors. First, the LKB1 protein expression scores were compared with RNA expression levels as determined by microarray profiling. Ordered logistic regression analysis demonstrated a positive relationship between protein scores and gene expression (n = 122; *p = 0.002*), observed as a positive upward slope on the plot shown in Fig. 5A. This is further evidence that the LKB1 antibody can faithfully detect the protein in clinical samples. Consistent with this interpretation, tumors with confirmed loss-of-function mutations showed lower average gene expression scores (*p = 0.059*, Wilcoxon rank sum test) (Fig. 5B). Likewise for protein expression scores, statistical and visual comparison of the two groups (mutation predicted to alter protein vs. no mutation, Table 2) showed lower protein expression in the mutation group; the plot is asymmetrical, and cases with mutations were skewed towards “0” scores (Fig. 5C; Fisher's Exact Test p = 0.04). Thus, although post-translational mechanisms may also contribute to LKB1 downregulation in tumors, statistically-significant relationships were observed between LKB1 protein scores and expression at the RNA level as well as mutational status. However, the number of cases with *LKB1* mutations was too small to assess relationships between types of mutations (e.g. premature stop vs. single amino acid substitutions) and LKB1 protein scores; such a determination will require larger studies. Next, we assessed the correlation between LKB1 and pAMPKα (Thr172) scores. A heat map was used for a graphical representation of the association of LKB1 and pAMPKα (Thr172) scores (Fig. 6). There was a significant association among cases which were scored for both markers (n = 122; Kendall's tau = 0.54, *p<0.001*), strongly arguing that both LKB1 and pAMPKα (Thr172) are interrelated biomarkers useful for interrogating LKB1 status in human clinical samples.

**Table 2:** Coding mutations and LKB1 scores.

Mutation(s)	LKB1 scores
exon 1: p.E70X	0 0 0
exon 2: p.Q123R	1 1 1
exon 4: p.Q170X	0 0 0
exon 4: splicing	0 0 0
exon 5: p.G242W	0 0 0
exon 5: splicing	1 0 0
exon 1: p.I88I exon 4: p.D194Y	0 0 0

In conclusion, we have shown through human cell lines and mouse *in vivo* models that endogenous levels of the LKB1 protein can be readily detected in multiple cell types in tissue sections. Furthermore, this assay can discriminate between different levels of LKB1 expression in human cancers. This assay, which employs a monoclonal antibody, should thus prove highly reproducible and easily adopted by diverse research or clinical laboratories for diverse investigations of the potential of LKB1 as a predictor of clinical outcomes in diverse human malignancies.
